# Geometric structure design of passive label-free microfluidic systems for biological micro-object separation

**DOI:** 10.1038/s41378-022-00386-y

**Published:** 2022-06-06

**Authors:** Hao Tang, Jiaqi Niu, Han Jin, Shujing Lin, Daxiang Cui

**Affiliations:** 1 Institute of Nano Biomedicine and Engineering, Shanghai Engineering Research Center for Intelligent Diagnosis and Treatment Instrument, Department of Instrument Science and Engineering, School of Electronic Information and Electrical Engineering, Shanghai Jiao Tong University, 800 Dongchuan RD, Shanghai, 200240 China; 2 National Engineering Research Center for Nanotechnology, Shanghai Jiao Tong University, 28 Jiangchuan Easternroad, Shanghai, 200241 China

**Keywords:** Microfluidics, Nanostructures

## Abstract

Passive and label-free microfluidic devices have no complex external accessories or detection-interfering label particles. These devices are now widely used in medical and bioresearch applications, including cell focusing and cell separation. Geometric structure plays the most essential role when designing a passive and label-free microfluidic chip. An exquisitely designed geometric structure can change particle trajectories and improve chip performance. However, the geometric design principles of passive and label-free microfluidics have not been comprehensively acknowledged. Here, we review the geometric innovations of several microfluidic schemes, including deterministic lateral displacement (DLD), inertial microfluidics (IMF), and viscoelastic microfluidics (VEM), and summarize the most creative innovations and design principles of passive and label-free microfluidics. We aim to provide a guideline for researchers who have an interest in geometric innovations of passive label-free microfluidics.

## Introduction

Microfluidic micro-object separation devices are referred to as delicately designed and fabricated devices, normally on the scale of micrometers or even nanometers; these devices are able to manipulate the path through which small particles pass. Separation, purification, and enrichment of biological microparticles are necessary steps before clinical and bioresearch applications^1^. For example, circulating tumor cells (CTCs) and circulating tumor DNA (ctDNA) isolation help early-stage cancer diagnosis^2–4^, and spore enrichment is a prerequisite of chemical analysis and production^5^. Microfluidic separation devices have established their reputation based on a reduced sample and reagent volumes, improved portability, significant sensitivity, and low cost^6^. Minute bioparticles, such as red blood cells (RBCs) and white blood cells (WBCs)^7^, CTCs^2,8–10^, exosomes^11,12^, DNA^13^, parasites^14^, bacteria^15,16^, and spores^5,17,18^, can be separated by microfluidics based on their size differences and other attributes. There are two types of microfluidic separation devices: active and passive^19^. The separation function of active microfluidic devices is given by a variety of external forces, such as magnetic^20^, electric^21,22^, acoustic^23^, centrifugation, and optical trapping^24^ forces, therefore bringing versatility to the system. However, drawbacks such as low throughput and unreliability come along with complexity^25^. For example, droplet-enhanced active micro-object manipulation achieves higher precision^26–28^, but its throughput is reduced considerably (260 cells/min)^29^. Passive microfluidics has advantages such as low cost and high throughput, which provides an alternative approach when active methods fail.

Passive microfluidic separation devices can also be classified into two types: labeled and label-free. Labeled devices contain functional molecules attached to a substrate or on the particles themselves. When particles in a fluid sample stream pass the substrate, they will be attached to the functional molecules. Functional molecules include aptamers, antibodies, and other proteins^20,30,31^. In some cases, magnetic beads are used as labels on biological micro-objects for separation^32–36^. In contrast, label-free microfluidic devices require no functional molecules, and their separation ability depends solely on fluid and particle dynamic properties and fluid-wall interaction properties inside the chip^37–39^. By designing precise structures of the walls and adjusting the inflow stream delicately, an ideal separation result can be obtained. Therefore, label-free microfluidics outperforms labeled microfluidics in terms of simplicity, reliability, and detection accuracy. Label-free microfluidics is composed of different schemes, such as deterministic lateral displacement (DLD), pinched flow fractionation (PFF), cross-flow filtration (CFF)^40^, hydrodynamic filtration, inertial microfluidics (IMF), and viscoelastic microfluidics (VEM). Note that the passive schemes mentioned above can also be integrated with active schemes to achieve better separation performance^41^. For example, dielectrophoresis forces help to reduce the critical diameter of a DLD device^42–44^. Previous works have been performed to summarize passive and label-free microfluidics^45–47^, but none of them have focused on the design laws of geometric structures of different schemes. Benefiting from its small size, low cost, and high cell viability, passive and label-free microfluidic technology has been widely applied in commercial devices. DLD commercial devices for CTC enrichment have achieved a high recovery of >95% and a high RBC and WBC removal rate of >99% (Nanocellect WOLF Cell Sorter). In addition, droplet technology has been used to assist in commercial cell separation devices.

**Fig. 1:**
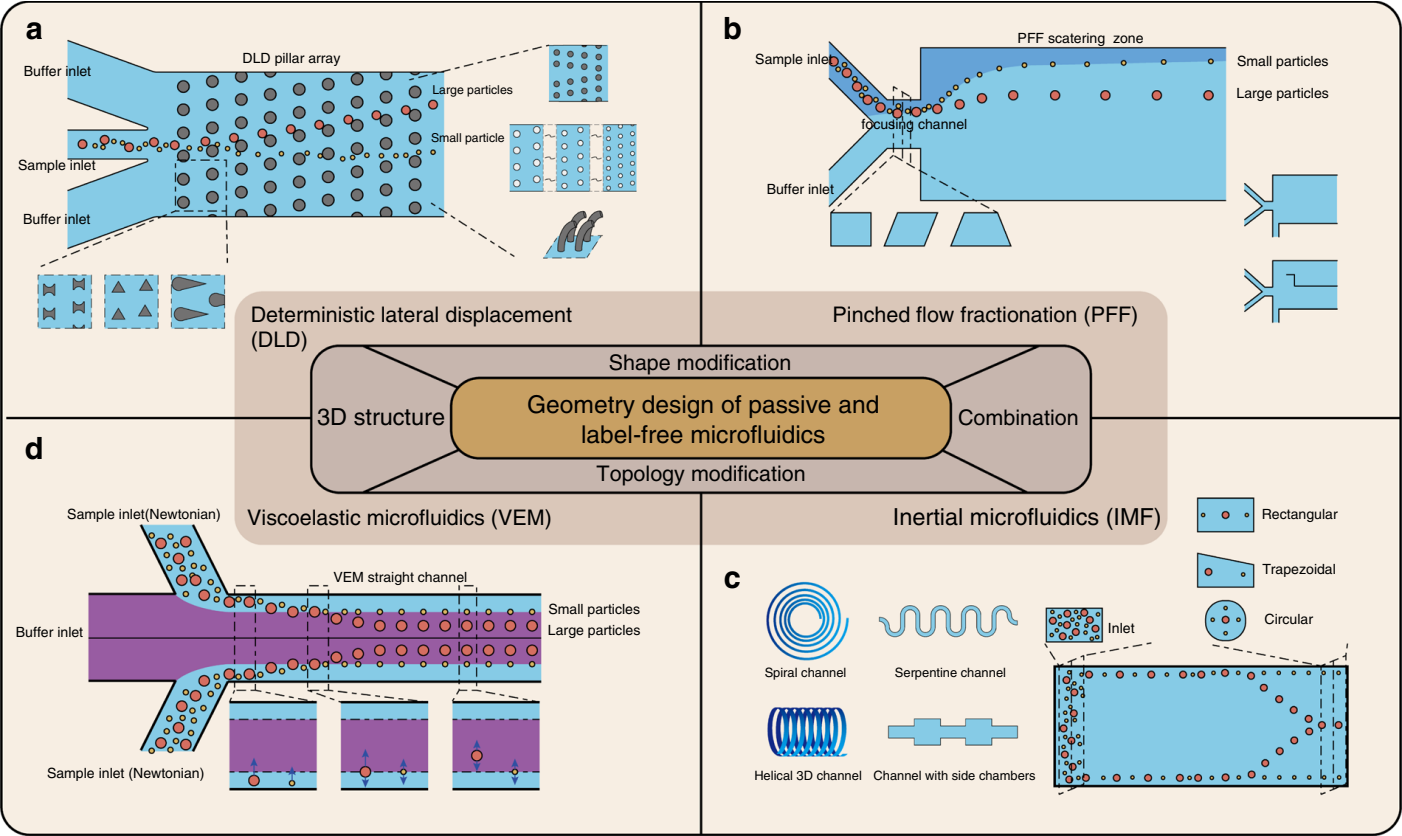
Geometry design of passive and label-free microfluidics. A brief illustration of the geometric design of passive and label-free separation microfluidics, including four main physics schemes: **a** DLD, **b** PFF, **c** IMF, and **d** VEM.

In this review, we discuss different geometry design methods for passive label-free microfluidic chips with a focus on the following physical schemes, namely, DLD, IMF, and VEM, and their combinations (Fig. 1). Other related schemes, such as CFF, PFF, and hydrophoresis, are also mentioned. We summarize the background mathematics and physics theories of each method, introduce their geometric structures and geometric design types, and weigh the pros and cons of each scheme. In Section 2, geometric design types of several different microfluidic schemes are discussed in detail. In Section 3, the combination methods of these geometric designs are introduced. In Section 4, a summary of and prospects for geometric designs are listed. We aim to prove that geometric design plays an irreplaceable role in passive label-free biological separation microfluidics and to provide a guideline for designing microfluidic geometries. The following aspects of microfluidic geometric design are mainly discussed in this review:

1. A comprehensive summarization of the geometric design of DLD, IMF, VEM, and other passive and label-free microfluidic schemes.
2. An instructive conclusion of geometric design principles for every microfluidic scheme.
3. The effectiveness analysis of every geometric innovation.

Table 1 summarizes some typical studies with creative geometric designs covered in this review and provides comprehensive guidance to readers.

**Table 1:** Summary of typical geometric designs in passive label-free separation microfluidic systems

Schemes	Geometry design types	Geometry design description	Particles to separate	Purity	Recovery	Throughput	Other promotions	Ref.
DLD	Pillar gap and size	Pillar gap variation	PS beads/RBCs	-	>95%(RBCs)	-	Increased throughput	^63^
		Pillar size variation	Fluorescent beads	-	-	-	New DLD displacement theory	^64^
	Pillar shape	Triangular pillar	Fluorescent beads	-	-	-	Reduced clogging increased throughput	^65^
			-	-	-	-	-	
		I-shape/L-shape pillar	-	-	-	-	Increased lateral displacement	^15^
		I-shape	PS beads/RBCs/E. coli	100%(RBCs)	-	-	-	^73^
		L-shape	RBCs	>99.7%(RBCs)	-	-	-	^74^
		Protrusion-curvature structure	CTCs	-	99%(CTC clusters)	-	-	^208^
		Notched pillar	RSCs	-	80%	20 μL/min	-	^77^
		Airfoil pillar	10-μm beads	-	75%	-	High throughput (Re = 51)	^80^
			15-μm beads	-	83%	-		
			20-μm beads	-	100%	-		
		Sieve-based pillar	Visualization beads	-	-	-	High throughput (100 < Re < 600)	^82^
		Sieve-based pillar	PS beads	-	-	120 μL/min	Reduced clogging	^81^
			WBCs	78 ± 14%	95%			
		Filter pillar	CTCs	99.995%	-	1 mL/min	Reduced clogging	^68^
		Topology-optimized pillar	2-6.5-μm beads	-	92.2%	-	Reduced clogging	^67^
	Combination of DLD arrays	Parallel mirrored device	Extracellular vesicles	-	50%	900 μL/h	Increased throughput	^84^
		Parallel mirrored device	Water-in-oil droplets	100%	100%	0.2 mL/h	Increased throughput	^86^
		Cascaded device	CTCs	>50%	>90%	12 mL/h	Multiple stage separation	^8^
		Parallel segmented device	0.6-1-μm beads	-	-	-	Increased dynamic separation range	^87^
	3D DLD	Gravity-driven 3D device	Nylon beads	≥89%	≥95%	-	-	^99^
		Sieve-based 3D device	785 µm beads	-	95%	2 mL/min	Increased throughput	^100^
		Revolved 3D device	60-μm beads	99.8 ± 0.5%	-	-	Increased throughput	^53^
			100-μm beads	98.7 ± 1.2%	-	-		
			150-μm beads	99.1 ± 0.4%	-	-		
	Simplified DLD	Single bumping column	4.8- and 9.9-μm beads	99%	99%	54 μL/min	Simplified structure/increased throughput	^102^
PFF	Drainage channel	Asymmetric outflow drainage channel	1.0-5.0-μm beads/RBCs	-	80% (RBCs)	-	Increased resolution	^153^
	Duplication	Duplicated focusing channel	0.5- and 1.5-μm PS beads	-	-	-	70% separation enhancement	^151^
	Focusing channel cross section	Parallelogram cross section	3-, 6-, and 10-μm PS beads	100%(10-μm beads)	-	-	-	^177^
IMF	Straight channel	two-stage straight channel	10- and 20-μm beads/CTCs	>90%	>99%	≥100 μL/min	-	^114^
		Straight channel with buffer inlets	19-μm beads	-	100%	-	-	^118^
			HeLa cells	98.5%	81.4%	-		
	Spiral channel	Triplet parallelizing spiral channel	MCF-7 cells	-	80–90%	80 ml/h	-	^131^
		Obstacle-based spiral channel	PS beads	-	99.8%	-	-	^128^
			MCF-7 cells	-	97.5%	-		
			HeLa cells	-	92.3%	-		
	Serpentine channel	Asymmetric serpentine channel	Fluorescent PS beads/RBCs	-	-	15,000 cells/s	-	^139^
		Serpentine channel with 3 outlets	2-m cyanobacteria	-	96.3 ± 0.3%	-	-	^142^
	Side chamber	Straight channel with chambers	RBCs	99.6%	-	-	-	^209^
			WBCs	91.0%	-	-		
	Combination	Serpentine channel after spiral channel	CTCs/WBCs/RBCs	93.60% (CTCs)	93.84%(CTCs)	-	99.992% blood cell removal rate	^158^
VEM	Straight channel	Sample-sheath flow channel	4.8-μm PS beads	-	-	20 μl/min	15-μm lateral displacement	^169^
		Newtonian and viscoelastic fluids	*Staphylococcus aureus*	>98%	97%	3.0 mL/h	-	^170^
			Platelets	-	100%	-		
Straight channel	Shear-induced diffusion	Sandwiched straight channel	PS beads	-	94.4%	6.75 mL/h	-	^180^
			Hep G2 cells	-	89.1%	-		
	Cross-flow microfiltration	Cross-flow membrane filtration	B. polymyxa	-	-	-	Extremely high throughput (Re >4000)	^181^
Combination	DLD/IMF	DLD array after IMF spiral channel	CTCs	92 ± 3%	-	5 mL WB/3 h	-	^98^
		Serpentine IMF channel after DLD array	CTCs	-	98.6 ± 4.3%	10^7^ cells/s	-	^192^
	DLD/VEM	DLD array with viscoelastic fluid	8- and 12-μm beads	-	-	-	Dynamic control of critical size	^196^
	IMF/CFF	Three-stage spiral focusing device	20-μm beads	-	99.99%	5 mL/h	-	^197^
			MCF-7 cells	-	90.4%			
			WBCs	-	97.97%			
	PFF/BFF	BFF after PFF	Beads/spores/eukaryotic cells	-	>90%(spores)	-	Large range of sample flow rates	^17^

*DLD* deterministic lateral displacement, *PS* polystyrene, *RBC* red blood cells, *CTC* circulating tumor cell, *RSC* retinal stem cell, *WBC* white blood cell, *PFF* pinched flow fractionation, *IMF* inertial microfluidics, *VEM* viscoelastic microfluidics, *CFF* cross-flow filtration, *BFF* branch flow fractionation.

## Different biological micro-object separation microfluidic schemes and their geometric structure designs

In this section, the geometric design of DLD, IMF, VEM, and other passive and label-free microfluidic schemes are discussed in detail. All of the geometric innovation principles of passive and label-free microfluidics can be categorized into the following four groups:

1. Shape modification. To change the shape of the primary structure of the scheme without changing its topology.
2. Topology modification. To change the topology of the primary structure. In a topology modification, boundary changes always occur, and sometimes new layouts form.
3. Combination. To combine several structures into one continuous device.
4. 3D structure. To extend the geometry modification into the third dimension.

We summarize these design principles and their subprinciples in Table 2. In the main text, we classify every geometry innovation into four groups, providing clear design guidance to readers.

**Table 2:** Geometry design principles of passive and label-free microfluidics

Designing principles	Subprinciples	Examples
Shape modification	Parametric optimization	DLD pillar size and gap optimization
		IMF/VEM rectangular cross section modification
	Shape optimization	DLD pillar shape optimization
		IMF channel direction modification
		IMF channel cross section shape optimization
Topology modification	Structural simplification	Simplified DLD
	Topology optimization	Topology-optimized DLD pillar
		Topology-optimized IMF channel
Combination	Combination within the same scheme	Cascaded/mirrored DLD array
		Duplicated PFF
		Combination of spiral and serpentine IMF channels
	Combination of different schemes	DLD array before an IMF focusing channel
		IMF channel before a DLD array
		DLD array with viscoelastic fluid
3D structure	3D structure	3D DLD
		3D IMF channel
	Top and bottom wall modification	Hydrophoresis

### Deterministic lateral displacement (DLD)

Deterministic lateral displacement (DLD) is a convenient separation tool that is widely used for cell separation, purification, and enrichment^48,49^ (shown in Fig. 1a). This technique was first proposed by Huang et al. in 2004^50^. The main idea of DLD is to separate particles by their sizes with the sample flow passing through a well-fabricated pillar array. The pillar array is deliberately tilted. Therefore, when the fluid flow encounters a pillar, bifurcation appears, and a certain number of streamlines are nearest to the pillar veer. As a result, small particles are able to veer along and travel in a zigzag mode, while larger particles whose diameters exceed a critical diameter (Dc) cannot veer and travel in a bumping mode. DLD can process particles ranging from nanometers^51,52^ (including exosomes) to hundreds of micrometers^53^. Label-free DLD has shown its potency to effectively separate cells and exosomes based on their sizes and deformability.

The most primitive model of DLD is made up of a circular pillar array with the same pillar space in both directions in the flow plane. Inglis et al. developed a theoretical model to determine Dc by assuming a parabolic velocity cross section at the inlet of a DLD unit^54^, providing a practical structure design theory for circular pillar DLD. The critical diameter can be calculated as:

$${{{\mathrm{Dc}}}} = {{{\mathrm{g}}}}\left( {1 + 2{{{\mathrm{w}}}} + \frac{1}{{2{{{\mathrm{w}}}}}}} \right)$$

$${{{\mathrm{w}}}} = \left[ {\frac{1}{8} - \frac{\varepsilon }{4} + \sqrt {\frac{\varepsilon }{{16}}\left( {\varepsilon - 1} \right)} } \right]^{1{{{\mathrm{/}}}}3}\left( { - \frac{1}{2} - \frac{{\sqrt 3 }}{2}{{{\mathrm{i}}}}} \right)$$

where g denotes the gap between pillars and ε denotes the ratio of the horizontal distance that each subsequent row is shifted. This method continues to work well in many recent studies, always giving a good approximation of the real Dc. Davis et al. later modified the theory by testing the particle separation behavior in devices with different row shift fractions and gap sizes^55^.

**Fig. 2:**
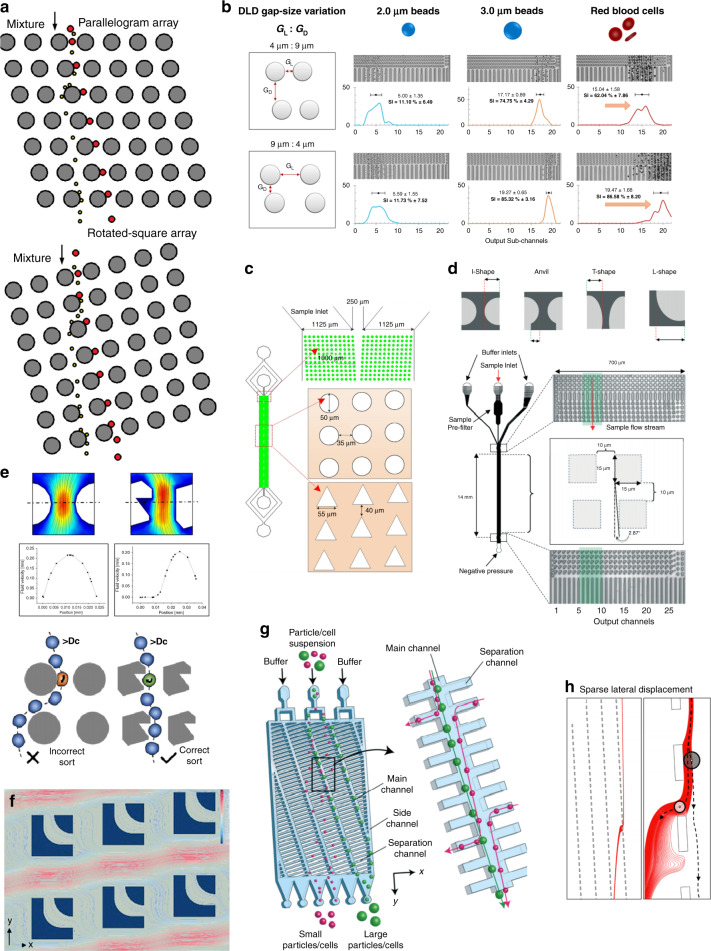
DLD pillar array design variations with pillar gap, size, and shape. **a** Comparison of the rotated square array and parallelogram array. **b** Adjusted pillar gaps of a DLD array^63^. **c** Triangular array for microalgae enrichment and purification^72^. **d** I-shaped, T-shaped, and L-shaped rotation-induced DLD separation^15^. **e** Notched DLD pillar array, which is able to induce shear stress, to sort and enrich retinal stem cells (RSCs)^77^. **f** Pillar topology variation. New boundaries emerge in a pillar^68^. **g** Size-selective sieve lattice structure with main channels and side channels^81^. **h** Simplified sieve-based DLD system^82^.

Except for the parallelogram pillar array, a rotated square array layout (Fig. 2a) is another feasible option^56^. The design principle of this change is classified as shape optimization. In a rotated square array, pillars are aligned as an orthogonal lattice, but the lattice direction forms a small angle with the flow direction. Large particles flow along the lattice direction forming a bumping mode, while smaller particles travel straight from inlet to outlet, forming a zigzag mode. Cerbelli designed a tilted square array to separate microparticles and studied the stochastic component of particles caused by diffusion^57^. Vernekar et al. investigated the performance of parallelogram and rotated square pillar arrays in cascaded structures^58^. These researchers proved that rotated square arrays are less prone to cause streamline deviation at the array connections. Reinecke et al. carried out the discrete element method (DEM) coupled with the Lattice Boltzmann method (LBM) simulation of suspended particles simulation of suspended particles inside a tilted square DLD array^59^. These authors also examined the streamline behavior when operating the DLD chip at different Reynolds numbers. The dependency of Dc on the particle density was also examined. Murmura et al. developed a transient DLD array that mimics classical chromatographic separation and is able to overcome the limitations of conventional stationary DLD arrays with tilted square arrays^60^. Biagioni et al. studied the 3D behavior of particles when passing a rotated square DLD array using a theoretical and numerical method^61^. Later, the same group investigated the unexpected trajectories of particles traveling in a zigzag mode in a rotated square array and proposed an electrostatic diffusion-advection model to interpret the phenomenon^62^. In the following part of this section, we mainly focus on geometric variations in the parallelogram pillar array, which is more widely applied in DLD design.

#### Modifications of DLD pillar gaps and sizes

Since a conventional circular pillar array cannot satisfy user demand in some circumstances, geometric modifications can be made to enhance performance. First, gaps between pillars in two different directions can be adjusted^36^ (parametric optimization). Zeming et al. demonstrated that an asymmetric DLD gap was able to achieve enhanced separation and throughput of red blood cells^63^. This model achieved a separation index greater than 95%, with no increase in flow resistance. Moreover, by setting a wider pillar gap laterally and decreasing the pillar gap along the flow direction, one can obtain a rather high throughput while simultaneously achieving better separation performance. The DLD pillar size is also a key factor, especially in devices where an altered zigzag mode is taken into consideration (parametric optimization). Kim et al. investigated circular pillar arrays with different pillar sizes^64^. These investigators suggested that a larger pillar size tilts the streamline, leading to a greater lateral displacement for the altered zigzag mode (Fig. 2b).

#### Modifications of the DLD pillar shape

The second geometry modification focuses on pillar shape (shape optimization). By replacing circular pillars with more complex geometry entities, the streamline pattern may change significantly. Triangular pillars have been proven to decrease the critical diameter^65,66^. A smaller critical diameter benefits the microfluidic device in many ways, such as reducing the clogging effect^67^, enabling a larger separation range, and maximizing the separation angle^68^. Much work has been done to alleviate the clogging effect and maintain high throughput^69,70^. Multiple agents have been applied in blood specimens to mitigate the clogging effect^71^, but pillar shape optimization has been showed to be a more generalized method. Wang et al. fabricated a triangular DLD array and a circular array for microalgae enrichment and purification for the first time and revealed that the triangular post array has a better performance over the circular array (Fig. 2c)^72^. Rectangular, L-shaped, and I-shaped pillars have also been well studied by both numerical and experimental methods, showing an even greater decrease in critical diameter^15,73–75^. Moreover, rotation-induced DLD separation has become increasingly prevalent in recent studies. I-shape and L-shape pillars are well known for their ability to rotate particles to adjust their travel mode, especially asymmetric particles such as RBCs, with their protrusions and curvatures^73^. Based on this phenomenon, Au et al. proposed an asymmetric pillar with protrusions and curvatures to rotate tumor cell clusters^76^. Gomis et al. designed a notched DLD pillar, which is able to induce shear stress, to sort and enrich retinal stem cells (RSCs) with a higher resolution (Fig. 2e)^77^. RSCs are always found in ciliary epithelium (CE) cells. A notched pillar has an advantage over a traditional circular pillar in that its void creates a low-velocity zone that allows the cell to rotate and reduces the deformation when a cell hits the pillar. This technique successfully separates RSCs from CE cells and outperforms the conventional FACS method. Sharped-edged obstacles may influence cell deformability, which is another key factor of DLD separation. Zhang et al. tested the performance of three different pillars (circular, diamond, and triangular) in RBC separation by simulation^78^. These researchers suggested that sharp-edged (diamond and triangular) pillars can induce a favorable mode of deformation compared to conventional circular pillars; therefore, they could serve as deformation sensors. Apart from polygon forms, some typical complex geometries have also been thoroughly studied. An airfoil-like pillar shows the capability to reduce cell deformation, therefore leading to a decrease in the critical diameter^79^. Dincau et al. developed another form of an airfoil pillar to decouple streamlines and vortex effects, allowing the chip to operate under high Reynolds number conditions^80^.

A new topology with boundaries emerging inside a pillar can be beneficial (topology optimization). Liu et al. proposed a novel filter DLD pillar array that can decrease Dc^68^. The filter pillar is composed of two individual parts, which can be seen in Fig. 2f. The two parts together form a filter channel, with a narrow inlet and a broad outlet. The filter channel is free for small particles to pass, while it blocks larger particles (diameter larger than Dc) at the same time. Furthermore, the filter channel exerts a downward drag force on large particles, thereby altering the streamlines, creating an asymmetric velocity profile, and decreasing the critical diameter. These authors also validated their structure in the cancer cell lines A549 and K562. By changing the gap and shape of pillars, the DLD pillar array can be reduced to a microsieve to guarantee a higher throughput. Yamada et al. designed a size-selective sieve lattice structure that can separate large cells from smaller cells (Fig. 2g)^81^ (shape optimization). This structure is composed of two types of channels intersecting perpendicularly: the main channels and the separation channels. The large cells are too large to enter the separation channels and always flow in the main channels, while smaller cells travel along the two types of channels successively. The width of the separation channels is set to 15 μm. This device achieved a high monocyte separation purity of 78 ± 14%. Dijkshoorn et al. presented a simplified sieve-based DLD system (Fig. 2h)^82^ (shape optimization). These authors visualized the flow lanes by CFD simulations and superimposed trajectory images of μ-PIV particles.

#### Combined DLD structures

A conventional DLD device always consists of only one pillar array. However, the combination of different pillar array geometries can achieve better separation results (combination).

**Fig. 3:**
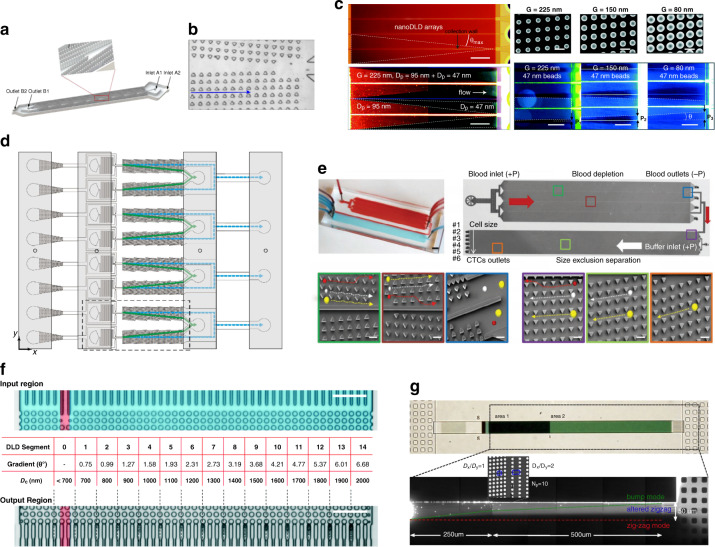
Different DLD arrays combined. **a** An integrated DLD device with a mirrored array structure to capture CTCs^2^. **b** Mirrored DLD structure for microalgae cell isolation^177^. **c** A mirrored nanometer DLD array to concentrate extracellular vesicles^84^. **d** A satellite-free emulsion droplet producer using parallel symmetric DLD arrays^86^. **e** Multistage separation DLD device to separate blood cells and CTCs^8^. **f** Segmented pillar array with multiple critical diameters to classify different-sized nanoparticles with an ultra-large dynamic range^87^. **g** Sequentially connected nanometer DLD arrays with different Dx/Dy ratios^64^.

A combination of two mirrored micropillar arrays is used to concentrate the bumping particles at the center of a microfluidic chamber. Conventional chambers are prone to send bumping particles to sidewalls, where streamlines deviate and clogging readily occurs. In a mirrored pillar array, however, bumping particles always migrate toward the chamber centerline. Jiang et al. developed a novel DLD device with a mirrored array structure to capture CTCs^2^, with a high capture rate of 83.3% (Fig. 3a). Feng et al. developed a mirror-symmetric array to concentrate different-sized beads at the center of the chip^83^. Their experiments were carried out with polystyrene spheres and leukemic T-cell lines. A mirrored layout is always combined with a parallel layout. Smith et al. developed a parallel mirrored nanometer DLD array to concentrate extracellular vesicles (Fig. 3c)^84^. Their device exhibited an excellent separation effect on particles with sizes ranging from 30 to 200 nm. These investigators also studied the intermediate mode where zigzag particles do not strictly follow the flow direction. Wang et al. proposed a prototype system using a mirrored DLD structure to isolate microalgae cells (Fig. 3b)^85^. These researchers tested the DLD array with two microalgal species. The separation efficiency of the targeted *Pyramimonas sp*. cells collected at the central outlet exceeds 85%, with a high maximum throughput of 200 μl/min. Later, the same group designed and fabricated a mirrored triangular microarray for the enrichment and purification of microalgae cells^72^. These authors showed that the triangular post array outperforms the conventional circular array with a maximum flow rate of 500 μl/min. Liu et al. developed a filter DLD array with a mirrored array structure to concentrate cancer cells in the center of the chip^68^. Their device achieved a high separation efficiency (>96%), high cell viability (>98%), high cell purity (WBC removal rate 99.995%), and high processing rate (1 mL/min). Tottori et al. designed a satellite-free emulsion droplet producer using parallel symmetric DLD arrays (Fig. 3d)^86^. This device is able to sort water-in-oil droplets with a Dc of 37.1 μm. High-throughput droplet generation (up to 0.2 ml/h) is achieved due to its parallel nature.

Cascaded DLD arrays are always used for multistage particle separation. Cascaded DLD devices consist of several different DLD arrays with different separation coefficients. There are different outlets for different-sized particles. Liu et al. proposed a multistage separation device to separate blood cells and CTCs (Fig. 3e)^8^. This device achieved an over 90% capture yield and over 50% capture purity. Zeming et al. developed a segmented pillar array with multiple critical diameters, which can classify different-sized nanoparticles with an ultra-large dynamic range (Fig. 3f)^87^. Kim et al. fabricated a nano DLD device with several arrays connected sequentially with different lateral permeabilities (Dx/Dy ratios) to validate whether an altered zigzag mode occurs as a result of the fluid streamline distortion caused by pillar arrays with different Dx/Dy ratios (Fig. 3g)^64^. These scholars also used microscale square arrays as the flow inlet and outlet. Liu et al. developed a cascaded filter DLD array to isolate and analyze CTCs^68^. Their device is composed of a parallel separation stage and a cascaded stage. The inclination angle of their pillar array increases gradually, which enables multi-Dc separation. Xavier et al. designed a cascaded mirrored DLD structure with two stages for primary human skeletal progenitor cell separation and enrichment^88^. In the first stage, small particles are altered to the channel walls by a DLD array with smaller Dc. In the second stage, Dc is designed to be smaller. As a result, large particles migrate toward the centerline of the channel, while small particles travel along the fluid flow direction near the sidewall following the zigzag mode. This design enables a larger lateral displacement. Kottmeier et al. proposed a DLD chip consisting of seven segments connected in a sequence to achieve a wider range of separation diameters^89^. The tilt angle of their DLD array varies from 1° to 6.7°, and Dc increases from 3 to 7.5 μm. Pariset et al. proposed a cascaded DLD separation device to successfully extract E. coli bacteria from blood samples spiked with prostate cancer cells^90^. The chip consists of two stages, each of which is formed by inlets, outlets, and a DLD array. The two stages are connected by a flexible chamber. The three components of the sample (blood cells, cancer cells, and bacteria) are separated with high efficiency. The depletion yield of cancer cells reached 100%. Arrays with different pillar shapes can be cascaded to achieve better separation performance. Wang et al. developed a device by connecting a triangular pillar array to a circular array^72^. This device promotes the efficiency of microalgae separation.

#### Other modifications of DLD

**Topology-Optimized DLD**: The design principles of the pillar shape modifications listed above can be summarized as follows:

1. Altering streamlines according to pillar shape/streamline relations;
2. Using protrusions and curvatures to rotate particles;
3. Reducing particle deformation by changing pillar shape.

**Fig. 4:**
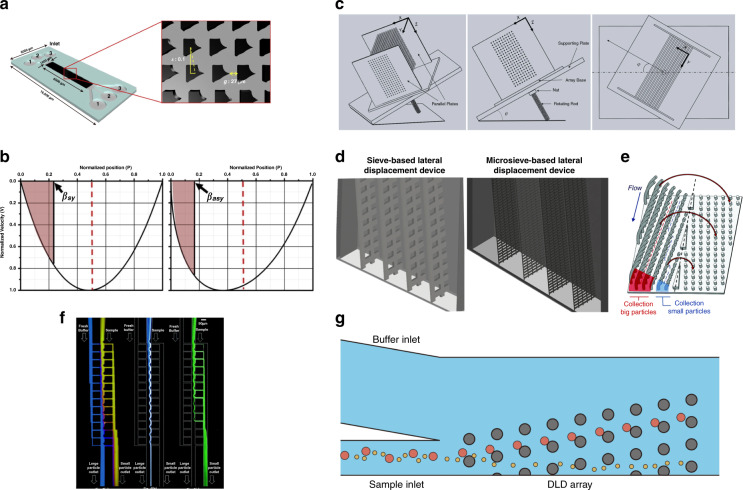
TO DLD, 3D DLD, and simplified DLD. **a** DLD pillar shape design by TO^67^. **b** The method of an asymmetric velocity profile^67,68,98^. The critical diameter decreases when the asymmetry of the velocity profile increases. **c** A gravity-driven 3D DLD array^99^. **d** Asymmetric 3D sieve-based DLD device to reduce the critical diameter^100^. **e** 3D mirrored DLD array to achieve high-throughput particle sorting^53^. **f** Single bumping column DLD device to shrink the DLD chip while maintaining its bumping and zigzag nature^102^. **g** Sparse deterministic ratchet structure. The number of pillars of this structure is reduced by half^104^.

However, all modifications can be achieved by individual physics hypotheses or theories, having some deficiencies, such as poor universality and extensibility. A more generalized approach is to use topology optimization (TO). TO directly connects the design goal to the topology structure by a predefined objective function. Theoretically, by properly designing an objective function, all kinds of separation performances can be achieved. TO is well known for its capability to create new boundaries via an optimization process. The applications of TO mainly lie in the area of solid structure optimization^91,92^. In 2003, Borrvall et al. pioneered the first fluid mechanics TO method in Stokes flow^93^. These investigators calculated an optimized reversed-flow structure in a 2D straight channel (shown in Fig. 4a), proving that TO is suitable for fluid geometric design. The TO of fluid-structure design works in various fluid schemes, including Newtonian and non-Newtonian environments^94^. For microfluidic devices, researchers have shown the feasibility of using TO for designing microvalves, micromixers, micropipes^95–97^, etc.

In the field of microfluidic separation devices, Hyun et al. developed a topology-optimized DLD chip with an asymmetric velocity profile to decrease the critical diameter to reduce the effect of clogging (Fig. 4b)^67^ (TO). Their experimental results illustrate that as the critical diameter decreases, clogging is significantly reduced. This is because gaps between the pillars must be wider to maintain a constant critical diameter. The method of asymmetric velocity profile has been showed to be an effective method of DLD pillar shape design; this method can be found in many studies (Fig. 4c)^67,68,98^. The optimization zone is designed manually. The gap between two design zones in 1 DLD unit is set as 15 μm. To describe the feature of asymmetry adequately, the objective function is set as a division of two integrations along the left and right half of the gap line, as shown in the following formula:

$${{{\mathrm{O}}}} = \frac{{\mathop {\int }\nolimits_0^{7.5} {{{\mathrm{vds}}}}}}{{\mathop {\int }\nolimits_{7.5}^{15} {{{\mathrm{vds}}}}}}$$

In the optimization process, the objective function O is maximized on the design zone to finally obtain a structure using the Darcy term TO method for fluid mechanics^93^:

$${\uprho}({{{\vec{\mathrm u}}}}\cdot \nabla ){{{\vec{\mathrm u}}}} = - \nabla {{{\mathrm{p}}}} + \nabla \cdot {\upmu}(\nabla {{{\vec{\mathrm u}}}} + \nabla {{{\vec{\mathrm u}}}}^{{{\mathrm{T}}}}) - {\upalpha}({\upgamma}){{{\vec{\mathrm u}}}}$$

where $\alpha (\gamma ){{{\vec{\mathrm u}}}}$ is the Darcy term. If γ = 1, the Darcy term disappears, and the equation above reduces to a normal Navier–Stokes equation, representing that there is no solid structure in a certain position. In contrast, if γ = 0, the TO structure appears. γ always converges to 0 or 1 in a successful iteration process. Finally, this structure maintains an up to 92% separation efficiency while greatly alleviating the clogging problem. However, the TO for DLD separation is not well explored due to the lack of flexibility of existing TO toolboxes, therefore leaving a broad research prospect.

##### 3D DLD

When the term DLD array is mentioned, we always refer to a 2D array that could force particles to travel in the horizontal plane and neglect the vertical dimension where gravity plays an important role. However, by extending the pillar array vertically to the third dimension, the performance of DLD can be further improved as the degrees of freedom of trajectories of particles increase (3D structure). Du et al. designed a 3D DLD device and showed its practicability to separate three kinds of beads with different diameters (Fig. 4d). These researchers indicated that the out-of-plane motion is dependent on the in-plane motion, which is an applicable phenomenon to improve performance^99^. Dijkshoorn et al. developed a sieve-based DLD device that achieved a lower pressure drop, lower risk of particle accumulation, higher throughput, and limited manufacturing difficulty (Fig. 4e)^100^. In this device, conventional pillar arrays are substituted by a sieve structure with aligned holes, which induces particle movement discrepancies in the third dimension. The effect of sieve size on critical diameters is investigated. Juskova et al. proposed a 3D high-throughput DLD structure with a critical diameter of 133 μm (Fig. 4f)^53^. This device is designed by extruding the pillars along an arc and is showed to be able to increase volume capacity and decrease shear rate. However, although the 3D device demonstrates a better separation efficiency, the structure is rather clumsy and is difficult to fabricate. As a result, the critical diameter of a 3D DLD device is significantly larger than that of a conventional planar DLD pillar array, which may prevent it from actual use. To overcome the fabrication obstacles, much work has been done. Juskova et al. developed a novel approach of 3D stereo-lithography^101^. The resolution and reproducibility are improved by applying direct control over the laser movement during fabrication.

##### Simplified DLD

Conventional DLD structures are complicated, which adds difficulty to fabrication and hinders accurate CFD simulation. To simplify the conventional DLD structure and to enhance device throughput, Liang et al. developed a single bumping column DLD device to shrink the DLD chip while maintaining its bumping and zigzag nature (Fig. 4g)^102^(TO). In their DLD chip, the bumping mode of large particles only appears in the middle column. The raised triangles at the center channel are used to enhance the bumping effect. This structure is able to separate small particles ranging from 5–110 μm at a very high throughput, which is over 10 times larger than that published in prior work^103^. Another simplified DLD structure is called sparse deterministic ratchet and is shown in Fig. 4h^104^(TO). This approach significantly reduced the conventional DLD lattice structure, leaving only half of the pillar array in use. Geometry and structure lines could be adjusted freely to the needs of users.

### Inertial microfluidics (IMF)

In microfluidic separation chips such as PFF or DLD chips, fluid inertia is always neglected. However, with increasing Reynolds number, inertia is no longer negligible, and some unexpected phenomena arise, which could benefit particle separation^105^. For example, the fluid velocity always increases as inertia becomes significant to bring about a much larger throughput; therefore, the separation efficiency increases. The geometric structure of inertial microfluidics always appears as a long channel. The Reynolds number of fluid flowing in this channel is high, always exceeding the Stokes zone to guide particles aloof from the streamline to form equilibrium positions. The long channel structure provides sufficient distance for particles to reach a stable condition. Modifying the geometric structure of the long channel has a profound effect on its separation performance.

The first phenomenon that increases the Reynolds number is inertial migration. Inertial lifting force brings about inertial migration in the direction perpendicular to fluid flow, as was first observed by Segre et al. in 1961^106^. As the Reynolds number exceeds the Stokes zone in a long circular cross-sectional straight pipe, the lifting force guides particles to migrate at distance from the centerline of the pipe. Meanwhile, a wall-induced repulsion force grows significantly when particles approach the pipe wall^107^, pushing them backward. As a result, particles reach an equilibrium point. The reason why there are forces guiding the particles to migrate laterally has not been entirely determined. However, there are many mature theories that can successfully predict the migration behavior of particles. For example, first deduced by Saffman in 1962, the sheer-induced Saffman force^108^ is a force that could lead a particle away from the channel centerline. It can be expressed as follows:

$${{{\mathrm{F}}}}_{{{\mathrm{S}}}} = 2{{{\mathrm{KVa}}}}^2\sqrt {\frac{{{{{\mathrm{u}}}}_{{{\mathrm{m}}}}\rho }}{{{{{\mathrm{vb}}}}^2}}}$$

where K is a constant, V is the relative velocity at which the particle lags behind the fluid, a is the radius of the particle, b is the radius of the tube, v is the kinetic viscosity of the fluid, *u*_*m*_ is the mean velocity, and ρ is the distance from the axis. Other lifting force theories include the Reynolds number-induced lifting force^109^, rotation-induced lifting force^110^, wall-induced lifting force^111^, etc. These forces together guarantee the appearance of equilibrium positions along the channel. In a long straight channel with a circular cross section, the equilibrium position points form a circle with a radius 0.6 times that of the channel cross section. In this section, we describe two major geometric modifications of IMF in detail: channel direction and cross-sectional modifications. After that, some other less applied modifications are introduced.

#### Modifications of the channel direction

As the fluid flows in a channel, the channel direction guides the fluid flow direction (shape optimization). Bending a long channel to form different patterns could lead to different separation performances. When traveling in a straight pipe, particles with different sizes migrate at different equilibrium points. Based on this principle of inertial migration, straight channels with modifications are developed for particle separation. A straight channel is well known for its structural simplicity and operational convenience^112^. The methods for modulating the equilibrium position include changing the cross section of the channel (diameter for a circular channel^113^, aspect ratio for a rectangular channel^114^) and changing the channel geometry in the flow direction^113^. Expansion-contraction zones can also help straight channels adjust particle trajectories^115^. Hur et al. demonstrated that single cells can be purified from cell clusters using inertial microfluidics in a straight channel based on different migration distances due to different particle sizes^116^. This separation channel is composed of two inertia regions: the focusing region and the separation region. Zhou et al. designed a multiflow inertial migration channel for CTC separation that can provide high purity (>87%) of separation^117^. Mach et al. developed a massive processing straight channel expansion device to separate RBCs and bacteria based on their size difference (Fig. 6a)^113^. In this work, an expansion region is designed to amplify lateral migration. Zhou et al. designed a straight channel with a rectangular cross section and a variant aspect ratio to separate rare cells in blood spiked with human prostate epithelial tumor (HPET) cells, achieving high efficiency (99%) and purity (90%)^114^. Dudani et al. developed a straight channel multiphase cell migration microfluidic device utilizing an inertial lifting force, which can send cells from one agent to another within milliseconds^118^. Wu et al. designed a bacteria-RBC separation device based on a combination of an asymmetrical sheath flow and proper channel geometry to deflect RBCs (~8 µm) and bacteria (~1 µm) with different lateral displacements by an inertia-induced migration force^119^.

Spiral channels are now commonly applied in inertial microfluidics. As a straight channel bends to a spiral, an asymmetry of the velocity in the cross-sectional plane arises, thus causing an interesting phenomenon called secondary flow^120^, also known as Dean flow. The inertia of the inner side of the spiral is larger, so the fluid there tends to flow outwards. Then, the fluid is pushed backward near the upper and lower walls due to the law of conservation of mass, forming two mirror-symmetric circular flows in the cross-sectional plane. The secondary flow is a key feature of spiral channels, which have a variety of applications, and this type of flow plays a major role in designing and manipulating the equilibrium position of inertial microfluidic channels. The most commonly used measure for Dean flow is the Dean number, which can be expressed as:

$${{{\mathrm{De}}}} = {{{\mathrm{Re}}}}\sqrt {\frac{{{{\mathrm{D}}}}}{{2{{{\mathrm{R}}}}}}}$$

where Re represents the Reynolds number of the flowing fluid and D and R represent the hydraulic diameter and radius of curvature, respectively. The Dean number denotes the ratio of inertial and centripetal forces to viscous forces, which provides an idealistic way to characterize the intensity of Dean flow^121^. Numerical and experimental studies have been carried out. For instance, Bayat et al. proposed a semiempirical Dean flow model to evaluate the average velocity of the flow^122^:

$$V_{De} = 0.031\frac{V}{S}De^{1.63}$$

With this formula, the velocity of the Dean flow with a Dean number under 30 can be precisely estimated.

The applications of spiral channels originate from a single curved channel. The spiral channel was first pioneered by Bhagat et al. in 2008^123^. Dong Hyun Yoon et al. designed a curved inertial microfluidics structure to separate particles based on size differences^124^. Later, Bhagat et al. proposed a 10-loop spiral particle focusing channel with a rectangular cross section by applying the Dean drag force and inertial lifting force^125^, and a focused particle stream was successfully observed by a laser detection setup. The same group then realized that the particle focusing principle might be able to guide the research of separation; thus, they further explored the separation application of this scheme. Three kinds of differently sized particles were injected into the spiral channel, and an over 90% separation efficiency was achieved^126^ at the outlet. Lee et al. developed a spiral structure, especially for bacteria-sized particles. In their study, three kinds of differently sized polystyrene beads were separated, and over a 97% efficiency was achieved^127^. An obstacle-based spiral channel for CTC separation was also investigated^128^ (Fig. 6b). Studies based on parallel channel and series connections have been carried out. Sun et al. reported a double spiral tumor cell separation channel, modified on a single spiral, with a collection rate of 92.28% of blood cells and 96.77% of tumor cells^129^. A multiplexed three-channel structure was also developed for circulating tumor cell (CTC) separation^130^. The idea of parallel channels can be applied in spiral channel IMF separation. Chen developed a triplet parallelizing spiral IMF chip for CTC separation^131^. The device is composed of three parallel spiral channels interconnected with each other. The author assumed that under the operation condition, the large cancer cells tend to migrate toward the centerline of the spiral channel. A circular spiral channel may further evolve into a rectangle and even into a 3D structure. Asghari et al. fabricated a 3D spiral structure by applying a “tape’n roll” method^132^. This method overcomes the fabrication difficulty of conventional 3D structures. In their work, both circular and rectangular spiral channels were investigated. Spiral channels with obstacles can achieve better performance in PS bead and CTC separation^128^. Elliptical spiral channels have also been applied in other utilities^133^.

Some researchers integrate 3D structures in spiral channels, forming 3D IMF devices. Palumbo et al. carried out a numerical study of another 3D inertial microfluidic channel with a helical structure (Fig. 6c)^134^. Geometric parameters such as the channel pitch, diameter, and taper angle were studied. Their numerical study shows good consistency with the experimental results. However, fabrication complexity has prevented the wider use of this structure. The 3D helical channel can be manufactured by 3D printing^135^. With this technique, one-step fabrication of manifold inertial channels can be created. Paiè et al. fabricated a 3D inertial channel for cell focusing^136^. Their channel is composed of tens of out-of-plane loops, which favors a compact parallelization of multiple focusing channels to promote throughput. Wei et al. designed a 3D helical IMF channel for ultrahigh-throughput single-cell sampling^137^. Their device was assembled by twining 360 μm tubing around 10 cm fused silicon tubing. Their helical tubing device achieved a single-cell sampling rate of 40,000 cells/min.

**Fig. 5:**
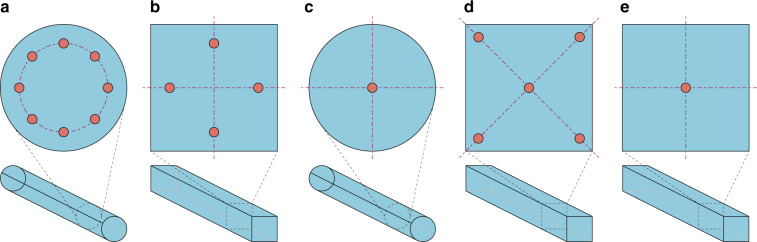
Different equilibrium positions of square and circular cross-sectional channels. Different equilibrium positions of square and circular cross-sectional channels working as **a**, **b** IMF and **c**–**e** VEM. The equilibrium position difference of square VEM channels (**d**, **e**) is caused by an inertial force. The number of equilibrium positions is reduced when the inertial force begins to play a non-negligible role in the VEM channel.

The serpentine channel is another direction modification scheme. In a spiral channel, the curvature remains the same or changes very slowly, and the channel bends in the same direction along the way. However, in a serpentine channel, the channel direction changes violently to increase the complexity of the state of fluid flowing inside the channel. In a spiral channel, curvature remains relatively stable, providing an ideal environment for achieving regular Dean flow. However, the effect is not the same in a serpentine channel, and a distorted Dean flow always appears. In addition, as thoroughly studied, the channel cross section plays a key role in defining the equilibrium position, and the most important cause of this phenomenon is symmetry. As depicted in Fig. 5a, b, when the channel cross section is circular (which means the highest symmetry), the number of equilibrium points is infinite, and all of them form a circle. As the symmetry weakens and the cross section becomes a square, the number of equilibrium points significantly decreases. The circular cross section is inadequate for separation performance because the focused pattern is one-dimensional (circular pipe shown in Fig. 5a), which is rather difficult to collect compared to 0 dimensions (points shown in Fig. 5b). Therefore, in some cases, breaking the symmetry of the channel may increase the focusing and separation efficiency^112^. This is one of the reasons why those modifications are made: Spiral channels with rectangular and trapezoidal cross sections break the symmetry along the flow direction, and the serpentine structure breaks the symmetry even more. Note that the serpentine structure can be qualified as another way to weaken symmetry because it introduces a violent angle change in the flow direction.

**Fig. 6:**
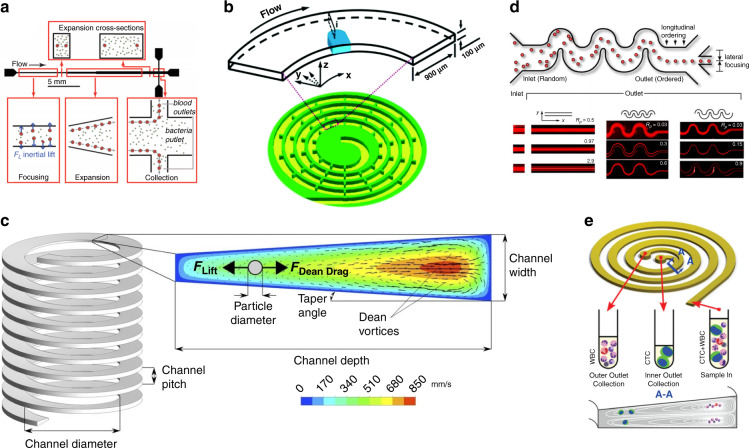
Geometry modification of the IMF channels. **a** Massive processing straight channel expansion device to separate RBCs and bacteria based on their size difference^113^. **b** Obstacle-based spiral channel for CTC separation^128^. **c** A numerical study of a 3D inertial microfluidic channel with a helical structure^134^. **d** Asymmetric serpentine structure to reduce the number of equilibrium points^139^. **e** Trapezoid cross-sectional channel to separate CTCs from WBC^158s^.

Free particle behaviors inside a serpentine flow were studied by Pedrol et al. by CFD. A homemade microfluidic device was used to verify the numerical results. With Dean inversions and abrupt gradient changes appearing frequently, the angular transition of flowing particles has been numerically studied^138^. Carlo et al. proposed an asymmetric serpentine structure to further reduce the focusing streams from 2 to 1 (Fig. 6d)^139^. Yin et al. carried out a comprehensive investigation of serpentine focusing channels and demonstrated several cross-sectional focusing patterns^140^. Xi et al. developed a microtube fabrication method and applied the method to various microfluidic structures, including serpentine channels, showing a 77–87% focusing efficiency^141^. Wang et al. developed another asymmetric serpentine structure as a novel microalgae concentration approach, aiming at low-cost, large-scale commercial manufacture. The device achieved a maximum recovery efficiency of 98.4 ± 0.2%^142^. Ducloué et al. focused on Dean flow and acquired the first Dean flow image using confocal microscopy. A comparison of the experimental results and reasonable numerical results exhibited a high matching degree^143^. Because of the complexity of serpentine channels, their theories and applications have not been comprehensively explored, leaving promising research prospects.

#### Modification of a channel cross section

The geometric structure of the spiral channel can be further modified with an eye on the channel cross section (shape optimization/parametric optimization). As shown in Fig. 5, the modification of the cross section could significantly improve performance. In a spiral channel, the Dean flow is produced solely by the spiral structure, and the channel cross section is always rectangular. However, Guan et al. showed that a trapezoidal cross section can create stronger Dean vortices and leads to a sudden transition of the equilibrium position, which is beneficial for higher resolution separation. These researchers showed that their trapezoidal cross-sectional channel could achieve an over 92% separation efficiency with ultrahigh throughput when separating 15.5 and 18.68 μm beads^144^; these results are much better than those of a channel with a conventional rectangular cross section. Cell separation applications of this trapezoid scheme appear in some major areas related to cell focusing and separation. A typical trapezoidal channel was proposed by Warkiani et al. to separate CTCs from WBCs. More than 80% of the cancer cells are isolated and detected at the outlet (Fig. 6e)^145^. Syed et al. used a channel with a trapezoid cross section to purify *Tetraselmis suecica* (lipid-rich microalgae) cultures from *Phaeodactylum tricornutum* (invasive diatom), with up to 95% of the target cells separated from the mixture observed^146^. Warkiani et al. developed another channel with a trapezoidal cross section to separate cells of different sizes to avoid clogging in membrane filtration, and high efficiency of 90% was achieved^147^. Kwon et al. developed a cell retention device applying a spiral channel with a trapezoidal cross section^148^. By adding two outlet channels at the inner and outer sides, the device achieved a cell retention rate of up to 97%. All of the examples shown above show the superiority of the trapezoidal cross section over the conventional rectangular cross section.

#### Other modifications

##### Side chambers

**Fig. 7:**
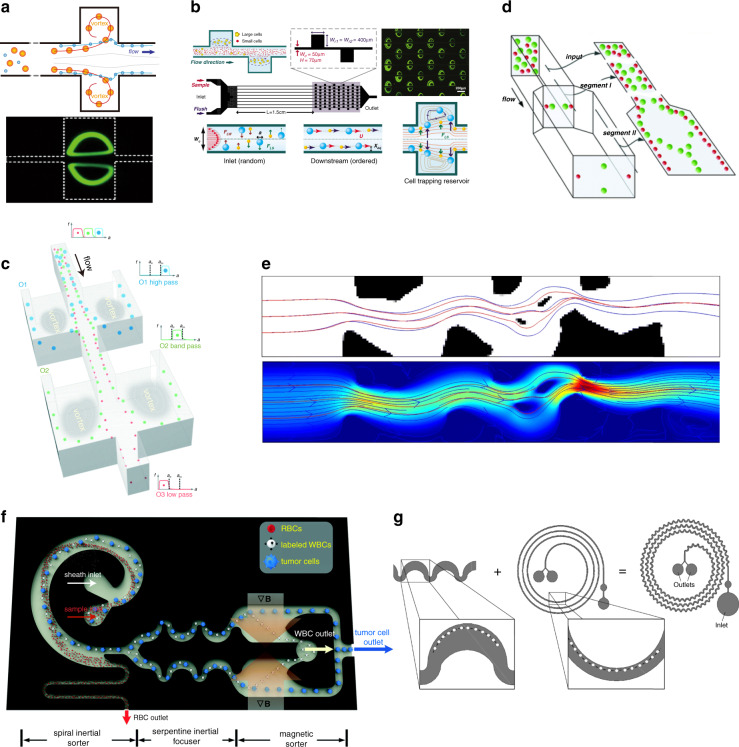
Inertial microfluidics based on side chambers. **a** The side chamber traps large particles inside, and smaller particles skip the chamber freely^149^. **b** Chambers are duplicated and parallel to solve the particle accumulation problem^150^. **c** Chambers with side outlets let trapped particles out^152^. **d** Modification of the IMF channel aspect ratio^114^. **e** Topology optimization method to design the IMF channel^156^. **f** CTC isolation device (3-stage) with a spiral separation channel before a serpentine focusing channel^158^. **g** A novel IMF structure obtained by superimposing spiral and serpentine channels ^159^.

Despite making use of Dean flow vortices to manipulate the equilibrium position by introducing a spiral channel and trapezoidal cross section, vortices can also be created by a side vortex channel (TO). Unlike the Dean flow vortex, a side vortex emerges in the plane parallel to the flow direction rather than in the cross-sectional plane. Side flow vortices are always much larger. This is because tilting particle trajectories to a new chamber zone requires a greater displacement than just manipulating equilibrium positions in the main channel. Zhou et al. carried out numerical and laboratory experiments and then showed the efficiency of a primitive particle trapping (isolation) chamber structure using a side vortex to separate particles based on their size differences^149^. Their structure is shown in Fig. 7a. A fluid mixture containing evenly scattered large and small particles is injected into the inlet. A long straight channel is connected after for inertia focusing. When the focused particle beam encounters the trapping chamber, larger particles are tilted toward the chamber and then trapped inside, while smaller particles skim freely over the chamber. The result shows that a threshold Reynolds number is the key factor in determining whether particles enter the trapping chamber. However, the structure has some severe deficiencies. First, the chamber lacks an outlet, resulting in a reduction in particle collection ability as the trapped particles accumulate. In addition, the separation performance of the chamber structure is highly dependent on sample concentration. Hur et al. designed a high-throughput microfluidic vortex structure^150^. We can see from Fig. 7b that the chambers are duplicated, which can partially solve the particle accumulation problem, and several channels are connected in parallel, which is an efficient way to improve throughput. This duplication method is similar to that of the previously mentioned PFF duplication^151^. Wang et al. proposed a multimodal separation side vortex structure with a side outlet added to the chamber, letting trapped particles out (Fig. 7c)^152^, which may solve the problem of particle accumulation. This structure is also shown to have high critical diameter tunability and flexibility. High critical diameter tunability is achieved by modifying the geometric structure of the chamber outlet to alter the flow resistance, similar to the drainage outlet of the PFF^153^. A similar study with a side outlet channel was carried out by the same group^154^. Raihan et al. recently proposed a low Reynolds number side chamber inertial microfluidic structure that could effectively separate 5 and 15 μm particles^155^. This device works at the Reynolds number, which is 1 order lower than traditional inertial microfluidics.

##### Channel aspect ratio

By modulating the channel aspect ratio, equilibrium positions can be altered. Several IMF channels with different aspect ratios can be cascaded to form a complex channel. Zhou et al. reported a channel with two different aspect ratios achieving high separation efficiency (>99%) and purity (>90%)^114^ (Fig. 7d). The modulation of the aspect ratio helps to reach complete separation.

##### Topology-optimized IMF

Topology optimization can also be implemented in IMF geometric design (TO). Andreasen et al. proposed a topology optimization (TO) method to design IMF channels (Fig. 7e)^156^. These investigators suggested that TO is feasible for particle trajectory and particle focusing IMF design. However, limited by computational ability, their optimization can only be applied two-dimensionally. Different from DLD, the geometry of IMF is simpler with fewer constrictions. Therefore, it is more possible for IMF to develop a real practical 3D TO design approach.

##### Combined IMF

Different IMF schemes can be integrated to achieve better separation and purification performance (combination). Tu et al. designed a parallelized IMF chip consisting of three channels^157^. These researchers designed the parallelized layout by an electrical circuit analogy. Huang et al. developed a rapid and precise cell separation device with three stages to isolate CTCs from whole blood (Fig. 7f)^158^. In this device, the first two label-free stages are designed for IMF sorting and focusing. The first IMF channel is designed to remove irrelevant RBCs, while the second serpentine IMF channel focuses on the remaining WBCs and CTCs for the final magnetic separation. This device achieved a high separation efficiency of 93.84% and a separation purity of 51.47% with undiluted blood. Sonmez et al. developed a novel IMF structure that was fabricated by superimposing two different schemes: spiral and serpentine channels (Fig. 7g)^159^. The two schemes are combined in such a way that their particle focusing positions are on the same side. An experiment carried out using 9.9 μm particles shows a significant enhancement of 14% over a spiral channel.

### Viscoelastic microfluidics

Viscoelastic microfluidic (VEM) devices work under the circumstances where viscoelastic forces play the major role and fluid flow is known as non-Newtonian flow, which is different from inertial microfluidics in 2.3^160,161^. Viscoelastic forces can bring about some unexpected phenomena leading to particle behavior change, which benefits the particle separation^162^. There are some important dimensionless numbers that can assess a viscoelastic flow. Apart from the Reynolds number, the most useful two of them are the Weissenberg number (Wi) and elasticity number (EI). Wi characterizes the ratio of viscous and elastic forces^163^, while EI compares elastic and inertial forces^164^. Wi and EI can be expressed by the following formulas:

$${{{\mathrm{Wi}}}} = \lambda \dot \gamma$$

$${{{\mathrm{EI}}}} = \frac{{{{{\mathrm{Wi}}}}}}{{{\Re} }}$$

where λ is the fluid relaxation time, $\dot \gamma$ denotes the shear rate, and Re is the Reynolds number. As the flow properties in IMF and VEM devices are different, particles flowing with the flow also behave differently. Such unique behaviors include viscosity thinning and extrudate swelling^165^. Particle focusing behavior is an important example of separation microfluidics. As mentioned before in inertial microfluidics, particles migrate to equilibrium positions in a long straight channel with a circular cross section, which is 0.6 times the channel cross-sectional radius. However, the equilibrium position of a VEM is at the center. Square and circular cross-sectional channels working as IMF and VEM are illustrated in Fig. 5. These different equilibrium positions provide another substitution for focusing and separation.

Because IMF and VEM only differ in fluid properties, their geometric structures and modifications are highly similar. The most representative structure of VEM is a long channel, similar to IMF. As some of the circular VEM have only one equilibrium position (others show five equilibrium positions), square or rectangular channels are always utilized for separation applications. Tunable parameters of VEM can be divided into two groups: fluid property-based and geometric structure-based. The ratio of inertial, viscous and elastic forces (Wi and EI) can be adjusted, while the channel can be winded to spiral, and the channel cross section can change from circular to rectangular, trapezoidal, etc.

**Fig. 8:**
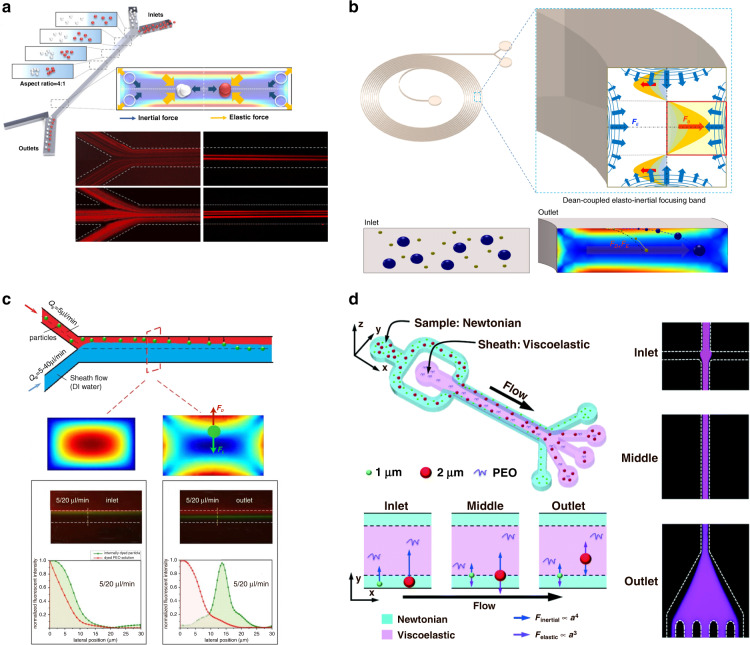
VEM geometric design. **a** A rectangular cross-sectional multiline separation structure^166^. **b** Spiral VEM channel to suggest that the Dean drag force and viscoelastic forces together alter the trajectory in a spiral channel^167^. **c** Particles traveling in between the sample and sheath flow^169^. **d** Co-flow of viscoelastic and Newtonian media devices, which achieves an over 90% separation efficiency and purity of 1 µm *Staphylococcus aureus* and 2–3 µm platelets^170^

There are several geometric structure modifications that are similar to those of inertial microfluidics (shape optimization): Yang et al. proposed a rectangular cross section multiline separation structure with varied aspect ratios. It is shown that the multiline separation effect is determined by the inlet geometry structure and aspect ratio (Fig. 8a)^166^. Particle equilibrium positions and normal stresses of 2:1 and 4:1 aspect ratios have been thoroughly studied, and the 4:1 structure exhibits a better separation result. Spiral structures can also be applied in non-Newtonian media to make use of Dean flow. Lee et al. showed that the Dean drag force and viscoelastic forces together alter the trajectory in a spiral channel (Fig. 8b)^167^. Numerical and laboratory experiments were carried out to analyze the particle performance under various Wi/De numbers and aspect ratios. Multiphase flow is often seen in VEM channels. Large particles are always observed to transition from Newtonian to non-Newtonian flow, while smaller particles always remain in Newtonian flow. Faridi et al. designed elasto-inertial microfluidics for bacteria separation from whole blood in two-phase flow^168^. Yuan et al. showed that particles could migrate laterally in sample-sheath flow at the Newtonian–non-Newtonian interface (Fig. 8c)^169^. The transfer efficiency is determined by the elastic force, channel length, flow rate, etc. With this principle, Tian et al. developed a co-flow of viscoelastic and Newtonian media devices that achieved an over 90% separation efficiency and purity of 1 µm *Staphylococcus aureus* and 2–3 µm platelets (Fig. 8d)^170^. In this structure, a viscoelastic flow is sandwiched between two Newtonian sample flows, and larger particles transform to viscoelastic flow while traveling.

### Other passive and label-free microfluidic schemes

#### Pinched flow fractionation

PFF has the simplest structure among all passive label-free separation methods, which was pioneered by Yamada et al.^171^. The physics principle of PFF is easy to understand: a buffer flow meets the sample flow in a microchannel, and large and small particles are pinched down to a side of the focus channel, which has a very small diameter, by buffer flow. Therefore, the lateral position difference of the particles is no longer negligible, as the diameters of the particles and the focused channel are comparable. A funnel-like zone is connected to the microchannel where the channel ends and therefore streamlines scatter and the lateral displacement of particles is amplified (Fig. 1b) (topology optimization). The funnel-like zone can be substituted by outflow channels in some cases, and inertial forces and rotation can be added to the system to improve separation efficiency^172^.

**Fig. 9:**
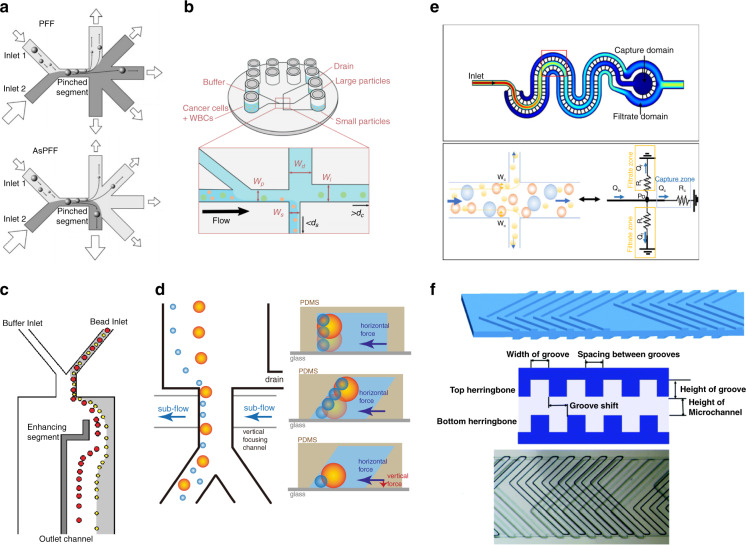
Geometric design of other passive and label-free microfluidics. **a** Asymmetric PFF structure with a drainage outflow channel^153^. **b** Cancer cell separation PFF structure with 2 separation outlets and 1 drainage^176^. **c** Duplicated PFF structure to amplify lateral displacement twice^151^. **d** PFF with parallelogram focusing channel^177^. **e** A serpentine pillar array design of the microfiltration device. **f** Double-sided herringbone microstructure for enhanced capture of rare tumor cells.

A variety of modifications can be made to improve the performance of an original PFF device based on different physical principles. Among them, we mainly focus on label-free passive ones; that is, changes are limited in geometric structures. The first modification is attributed to flow resistance. Note that even if large and small particles are separated because of streamline scattering, the original device is ineffective, as half of the funnel-like zone is unused, resulting in a non noticeable lateral displacement. A reduction in flow resistance at the buffer flow side helps to tilt streamlines and therefore to promote separation efficiency. The embodiment of decreasing flow resistance includes an increasing outflow channel diameter and a decreasing outflow channel width^153^. This kind of new PFF is named asymmetric PFF (AsPFF) (Fig. 9a) (shape optimization).

Further studies on AsPFFs have been carried out. One of the most widely applied numerical theories of PFF analysis is Lattice-Boltzmann simulation^173,174^. Based on this theory, the relationship between separation performance and the inflow and outflow ratio was comprehensively discussed by Ma et al.^175^. A new structure was promoted by Pødenphant et al., where the funnel-like zone is substituted by different channels to accommodate cancer cell separation (Fig. 9b)^176^ (shape optimization). There are three outflow channels in this structure, two of which are separation outputs, while the other is drainage to enable the asymmetric flow. An up to 90% separation efficiency is achieved with this structure, and the influence of cell deformability is discussed.

The next modification is achieved by duplicating the functional zone to enhance efficiency. Asger Laurberg Vig et al. proposed an enhanced PFF structure by duplicating the microchannel/funnel-like zone structure to amplify the lateral displacement twice (Fig. 9c)^151^. The result shows that amplification of separation of up to 70% was achieved.

The last modification is on a microfocusing channel (Fig. 9d)^177^, which aims to strengthen the focusing effect and lateral separation (shape optimization). By changing the rectangular cross section of the channel of AsPFF (there is a drainage in the funnel-like channel) to a tilted one and adding a vertical focusing channel, the separation distance of particles is increased from (*r*_*L*_–*r*_*S*_) to (*r*_*L*_–*r*_*S*_)/tan(*θ*/2). The new structure was found to be 11.6 times better than that of a conventional AsPFF. Later, the same group developed a similar device to separate spherical and disc-shaped particles^178^. The shape of the cross section of the microfocusing channel was changed to a trapezoid. The new device showed 2.3-fold and 2.6-fold improvements in separation for model particles. However, further use of the PFF structure is limited due to its small throughput caused by the focusing channel.

#### Microfluidic schemes with straight channels

Benefiting from its simplicity, a (modified) straight channel is the most widely applied structure in microfluidic geometric design. With attached structures such as side channels or side chambers, straight channels could work as microfluidic separation devices in different ways. In Part 2.3 and Part 2.4, the geometric design of two typical microfluidic schemes with straight channels are described in detail. However, despite IMF and VEM, there are several other microfluidic schemes that use straight channels.

In most separation studies, especially with blood, whole blood is pretreated with many trivial steps to obtain diluted, chip-processable samples. One of the major reasons to dilute blood is that whole blood contains too many cells so that particle–particle interactions cannot be neglected. Shear-induced diffusion (SID) provides an alternate way to separate cells in whole blood using particle–particle interactions without dilution. The basic theory of SID has not been thoroughly explored, but some progress in particle–particle interactions has been made^179^. Zhou et al. developed a straight channel with a rectangular cross section that achieves cell separation from the whole blood^180^. This device requires only sheath flow to form a sandwich fluid configuration without dilution. These authors also achieved an extremely high separation throughput, up to 6.75 mL/h, higher than the throughput achieved by inertial microfluidics.

CFF is another microfluidic technique that can work in a straight channel. Unlike conventional filtration approaches with dead ends, sample fluid flows tangentially to the filter structure (membrane or side channels)^181^. The small particles are filtered into side channels, leaving large particles in the main straight channel. This structure alleviates the clogging problem as large particles that are not able to cross the filter are washed away. Aran et al. designed a CFF device to extract blood plasma from the whole blood^182^. This device is showed to be effective, with a plasma protein recovery rate of over 80% and a low level of biofouling on the filter membrane surface during a long experimental period (over 4 h). A CFF device composed of serpentine pillar lines has shown its ability to separate PS beads with sizes of 10 μm and above, with capture efficiencies of ~95 and 85%, respectively (Fig. 9e)^183^.

#### Modification of bottom and top walls

In a conventional microfluidic channel, the bottom and top walls are flat. However, different structures can evolve at the bottom and top walls, and unexpected beneficial phenomena emerge. Hydrophoresis is a groove-based separation method that achieves high Reynolds number microparticle separation. Hydrophoresis devices are normally composed of a slanted groove array. The size-based separation effect of hydrophoresis is realized by secondary flow caused by a slanted groove array and groove–particle interaction. Geometric parameters of grooves, such as the width of the channel and the aspect ratio of grooves^184^, have been investigated, proving that an increase in the channel width helps the transformation from separation mode to focusing mode^185^. Other groove geometry innovations, such as V-shaped herringbone grooves, have been shown to be effective in cell focusing^186^. Hydrophoresis can also be combined with labeled microfluidics to enhance the separation performance of cells^187^. The herringbone structure is always used in micromixers^188^. Moreover, cell separation applications of herringbone grooves have been published. Hyun et al. proposed a reduced-deviation-flow herringbone structure for cell concentration, which achieved a recovery efficiency of 98.5%^189^. Wang et al. designed a microfluidic chip with double-sided herringbone microstructures to capture rare tumor cells (Fig. 9f)^190^. After geometry optimization, their device achieved a 94 ± 4% rare tumor cell capture efficiency from whole blood. Last, the combination of structures with different 3D characteristics is another instructive design direction. Separation experiments using cascaded DLD arrays with different chip heights were conducted for parasite separation^14^.

## Combination of different geometric structures

In general, higher performance could be reached by integrating several geometric structures with different physics schemes (combination). From the designations of the four schemes discussed above, PFF and DLD are named after particle and fluid behaviors, which are determined by fluid-wall interactions, and therefore their geometric structures cannot be modified too much. In contrast, IMF and VEM are named after fluid properties, so their geometric structures can be changed freely as long as a certain fluid property is in use. This nature enables the combination of the former and the latter two schemes.

**Fig. 10:**
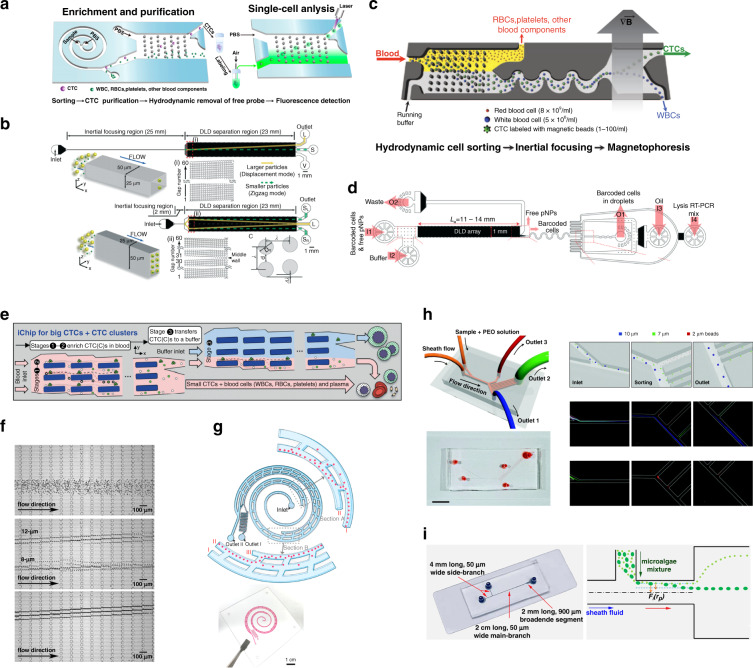
Combinations of different geometric structures. **a** Integrated microfluidic device for CTC separation with two stages: triangular pillar array after a spiral IMF channel^98^. **b** Sheath-free DLD system with a 25 mm rectangular straight IMF channel serving as a particle focusing stage^191^. **c** Inertial focusing-enhanced CTC capture chip with a crude DLD separation stage before an IMF serpentine focusing stage^192^. **d** Single-stream Drop-seq-derived method to screen barcoded pNPs against CD8+ T cells^193^. **e** Microfluidic device named a nonequilibrium inertial separation array (NISA) composed of rectangular islands, which uses inertial lift forces to substitute for post bumping forces in a conventional DLD device^103,195^. **f** Viscoelasticized force-enhanced DLD device^196^. **g** Integrated three-stage microfluidic device for cell concentration and sample volume reduction^197^. This device integrates IMF and CFF. **h** PFF/BFF (branch flow fractionation) fungal spore passive sorting device^17^. **i** Inertia-enhanced PFF for microalgae separation ^198^.

Inertial microfluidics is well known for its rapid separation and simple structure. As a result, IMF channels could be applied as a crude sorting step before slow but more precise steps. By connecting the IMF channel and DLD pillar array as a sequence, better separation results could be achieved. Pei et al. fabricated an integrated microfluidic device for CTC separation with two stages: a triangular pillar array after a spiral IMF channel (Fig. 10a)^98^. The first stage employs a rectangular cross-sectional spiral channel to focus CTCs and a small portion of WBCs with high throughput and sends them into the second DLD stage. The triangular pillar array carries out CTC sorting with higher purity. As a result, this device achieved high throughput and high purity (92 ± 3%) CTC sorting. Zeming et al. proposed a reticulocyte separation method from erythroid culture using IMF and DLD^74^. This method showed a significant improvement in cell sorting. These scholars also compared the separation efficiency of circular and L-shaped pillars, and the findings indicate that L-shaped pillars exhibit a better separation resolution and a larger lateral migration angle. IMF can also be used as a focusing stage before DLD separation, substituting conventional sheath flow. Tottori et al. presented a sheath-free DLD system with a 25 mm rectangular straight IMF channel serving as a particle focusing stage (Fig. 10b)^191^. Separation experiments were carried out with fluorescent polymer particles with diameters of 13 and 7 μm. This device demonstrates a very high capture efficiency of 99%.

The DLD array can also be applied as the first stage, followed by a postprocessing IMF channel. Ozkumur et al. developed an inertial focusing-enhanced CTC capture chip, which is termed the “CTC-iChip” (Fig. 10c)^192^. In this device, the first two label-free steps show a typical combination of DLD and IMF. The first DLD stage captures nucleated cells, mainly CTCs and WBCs, from whole blood. The second IMF stage aligns nucleated cells using a serpentine channel. This is to minimize the external magnetic field for cell deflection. The aligned and prelabeled nucleated cells are injected into the third magnetic separation stage, where CTCs are extracted from WBCs. Their device is able to sort CTCs from whole blood with a high throughput of 10^7^ cells per second. Alphonsus et al. proposed a single-stream Drop-seq-derived method to screen barcoded pNPs against CD8+ T cells (Fig. 10d)^193^. The cell sorting and focusing part of their device is divided into two stages. The first stage is a DLD array, and the second stage is a serpentine inertial channel to focus cells for droplet production.

The principles of DLD and IMF can be combined in a more direct way; specifically, a device can be fabricated with a DLD structure and IMF theory. Under the guidance of this theory, new pillars are designed to accommodate high Reynolds number inertial flow. Aghilinejad et al. studied the cell trajectories in DLD pillar arrays with different shapes as the Reynolds number increased^194^. These researchers also investigated the cell deformation and the cell-post interaction. Mutlu et al. fabricated a microfluidic device referred to as a nonequilibrium inertial separation array (NISA), which uses inertial lift forces to substitute for post bumping forces in a conventional DLD device (Fig. 10e)^103,195^. In their structure, DLD pillars are replaced by NISA rectangular islands, and 3–4% of the total flow is siphoned to the next island, forming a flow pattern similar to the zigzag mode of conventional DLD arrays. These authors attempted to use their device in separating RBCs and WBCs and demonstrated a high WBC yield of 95.7 ± 0.6%. Because of the IMF nature of the device, the chip could process blood samples at very high throughput, ~3 ml/min, or ~300 million cells per second. Long-term experiments were also carried out to show that clogging does not readily occur in the device. Later, the same group applied this structure in separating CTCs^195^. The blood cell removal test showed an excellent result, leaving 0.01% of leukocytes alongside CTC clusters in the final product.

The DLD pillar array can also be modified by changing the Newtonian sample fluid to a viscoelastic fluid. Li et al. utilized viscoelastic forces in a DLD device for the first time (Fig. 10f)^196^. The authors revealed that Dc can be tuned by changing the viscoelastic nature of the fluid. The Weissenberg number (Wi) was applied to evaluate the viscoelastic level. As Wi increases, the elastic lifting force plays a more significant role. This force pushes the particles away from the pillars and leads to a decrease in Dc, resulting in a particle trajectory change from the zigzag mode to the bumping mode. The DLD array was modified to accommodate a viscoelastic fluid by increasing the pillar gap along the fluid direction. Although Dc increases as the pillar gap is widened, the viscoelastic effect could easily cancel this slight variation. These researchers tested the dynamic control of Dc over 8 and 12 μm spheres by changing Wi.

The IMF channel can be combined with CFF to achieve higher separation efficiency. Xiang et al. designed an integrated three-stage microfluidic device for cell concentration and sample volume reduction (Fig. 10g)^197^. Similar to Chen’s device^131^, this structure consists of three spiral channels, which are interconnected by many CFF channels. As shown in Fig. 10g, this device achieves a high-fold cell concentration by repeatedly performing CFF after IMF focusing on different channels. Under this method, an extremely high cell concentration fold of 1100 is accomplished.

When combined with other geometry schemes, the performance of PFF can be promoted. Park et al. proposed another structure that integrates PFF and BFF (branch flow fractionation) (Fig. 10h)^17^. Their device was used for the passive sorting of fungal spores. In this structure, the BFF zone is connected to the PFF zone. The two connected zones separate particles into three groups: small waste, fungal spores, and large waste. This method achieved a high separation efficiency over applying PFF alone. Wang et al. developed an inertia-enhanced PFF that outperformed conventional PFFs in terms of accuracy and efficiency (Fig. 10i)^198^. The experimental results show that the separation efficiency is better as the Reynolds number increases. This device was able to separate microalgae with a high recovery rate of 90% and a purity of 86%.

## Numerical methods for geometric design

Numerical methods have been commonly applied in passive and label-free microfluidics. The most widely used numerical tool in passive and label-free microfluidics is computational fluid dynamics (CFD). CFD numerical experiments can be performed using many CFD tools, such as ANSYS fluent, COMSOL Multiphysics, and OpenFOAM. First, the velocity field inside a microfluidic device is always calculated to ensure the desired separation effect produced by the predesigned structure. When designing a DLD device, a single fluid field in a DLD unit is calculated because of the periodic nature of the DLD array. Zeming et al. designed a DLD array with varied pillar gaps by using a velocity field calculation^63^. Bhattacharjee et al. designed and analyzed an optimized microfluidic DLD channel for the isolation of CTCs by calculating the fluid field^10^. Based on the calculated velocity field, other derived analysis tools have been proposed. Kim et al. designed a DLD particle trajectory analysis method called a recurrence map^64^. The recurrence map is computed by streamlines connecting the inlet and outlet. The shape of the recurrence map determines the characteristics of a particle trajectory traveling in a certain DLD layout. The working fluid Re region of DLD numerical methods is Re«1, which is called the Stokes region, where the advection term is completely neglected. This is the same as other laminar-flow-based methods, such as PFF. CFD can also be applied in high Reynolds number fluids, such as an IMF channel. A number of numerical studies have been carried out to investigate guiding forces in IMF channels, such as the Saffman force^199^, wall lifting force, and Dean flow force^120^. Sun et al. designed a double spiral channel for cell manipulation assisted by numerical analysis of Dean flow^129^. Shiriny established a numerical model of a spiral IMF channel for the Dean flow profile with different Reynolds numbers^200^. The serpentine channel is also modeled in a 2D model^138^. Palumbo et al. carried out a numerical study of a 3D helical IMF channel^134^. Other 3D simulations, such as the V-shaped groove structure, have been shown to be effective and helpful in assisting geometry optimization^189^. However, because the flowing nature becomes complicated as the Reynolds number increases, a numerical study of complex 3D structures becomes difficult.

Because almost all microfluidic devices are made for microparticle manipulation, particle tracing has been widely applied in many studies. In a DLD or PFF device where inertia is often neglected, the Stokes drag force is always the only force that should be considered. The Stokes drag force is calculated by the particle mass, particle size, fluid viscosity, fluid density, and velocity field. In a DLD array, the bumping mode could be successfully modeled by the Stokes drag force combined with particle-pillar interactions. Kim et al. carried out a particle tracing numerical experiment using OpenFOAM^64^. In the IMF channel, where migration forces become complicated, particle tracing is much harder. Note that almost all particle tracing methods neglect the velocity field disturbance caused by particles. More precise simulation results can be obtained if the particle-water effect is considered. However, these multiphase techniques, such as moving meshes, are computationally costly and only suitable for extremely precise applications.

Cell deformation is a key factor that should be considered when guiding forces are intense. In a DLD array, cells always deform when they collide with pillars, leading to an unavoidable decrease in effective diameter. Khodaee et al. carried out a numerical experiment focusing on cell stress and deformation^201^. Chien et al. investigated the cell deformation behavior of erythrocytes in a 3D model^7^. Even though the existing models show the feasibility of modeling cell deformation, some important factors, such as membrane rigidity, membrane viscosity, and 3D velocity field, have not yet been taken into account. The numeric models of cell deformation are still primitive and need to be further explored.

## Conclusions and outlook

The enduring appeal of passive and label-free microfluidic particle separation is rooted in its simple structure, high resolution, and high throughput. Due to these advantages, passive and label-free microfluidic devices are now widely used in applications ranging from cell focusing to cell separation. Recently, label-free microfluidics has been discussed in detail in the previous works^202^. Geometric structure, fluid features, and particle properties are key factors that should be considered prudently in a design. Among all these factors, geometric design plays the principal role. With tremendous technological advances in microfabrication, numerous microfluidic structures have been designed and fabricated in recent years. This review summarizes geometric innovations of several microfluidic schemes, including DLD, IMF, VEM, etc. To the best of our knowledge, this is the first time that passive and label-free microfluidic geometric design has been discussed in detail and comprehensively, covering methods, structures, advantages and disadvantages, and the development of the geometric design of different microfluidic schemes.

Because of the complexity of the microfluidic device fabrication process, it is necessary to simplify the experimental design process. When designing a specific device, one can choose numerical methods, including physics analysis, parametric sweep, and structural optimization algorithms, before actual experiments are carried out. With the rapid development of numerical calculation (CFD) methods such as finite element analysis and the finite volume method, geometric structures of microfluidics can be designed and optimized precisely in simulation software^75,203–205^. Useful software for CFD simulation includes COMSOL Multiphysics, ANSYS fluent, uFlow (for inertial fluid flow and pillar sequence design)^206^, etc. Open-source CFD software OpenFOAM has also been applied by some researchers when more advanced simulations are needed. A well-designed geometric structure leads to a reliable computer simulation.

Designing theories of geometry are varied, and all geometric modifications are made to improve the performance of separation, purification, and enrichment. Despite the lack of general mathematical theories guiding all the forms of geometric design, designing theory can be logically concluded based on the physics backgrounds of each microfluidic scheme. For example, the channel cross section and scattering zone in PFF devices, pillar gap, pillar sizes, pillar shape, and array dimension in DLD devices, and channel cross section, channel direction, and side-channel structure in IMF devices can be modified. All of the geometry innovation principles can be categorized into the following four groups: shape modification, topology modification, combination, and 3D structure. These four main groups together guide the design of passive and label-free microfluidics. Some design methods are versatile and powerful in designing microfluidic geometries with different schemes. For example, duplication is used for performance enhancement^150,151^. TO has shown to be promising in both DLD and IMF design^67,156^. Parallelization is good for clogging reduction^86,150^. The drainage channel can be applied in both PFF^153^ and IMF^152^ chips. As computer technology is developing, intelligent and automatic design methods have become increasingly prevalent. Artificial intelligence (AI) technology may also be applied in a microfluidic geometric design in the future^207^.

The geometric design of passive and label-free microfluidics is not limited to the existing developments of this review. In the future, more advanced geometry innovations are bound to appear. Geometric design is likely to develop following the three paths. First, the design principles will merge with the development of applied mathematics. This is the reason why TO is discussed in detail, as TO points out a possible way to synthesize all designing theories. In addition to TO, AI technology is another promising field that may unify design theories. Second, geometric design is heading in the direction of high automation. This trend is accelerated by the synthesis of different design theories. Last, with the rapid development of microfabrication technology, 3D structures will greatly improve the performance of conventional 2D microfluidic chips.

In summary, passive and label-free separation microfluidic systems benefit from geometric design. With the development of microfabrication, micro/nanocharacterization, and measurement technology and computer technology, geometric design is developing by leaps and bounds.
